# Editorial: Microglial Polarization in the Pathogenesis and Therapeutics of Neurodegenerative Diseases

**DOI:** 10.3389/fnagi.2018.00154

**Published:** 2018-05-24

**Authors:** Yu Tang

**Affiliations:** National Clinical Research Center for Geriatric Disorders, Xiangya Hospital, Central South University, Changsha, China

**Keywords:** neurodegenerative diseases, neuroinflammation, microglial polarization, M1 microglia, M2 microglia, M1/M2 switch, aging

Neurodegenerative diseases are a clinically heterogeneous group of disorders characterized by the progressive degeneration of neurons in both central nervous system (CNS) and peripheral nervous system (PNS), among which microglia-mediated neuroinflammation is one of the shared prominent features. Microglial polarization appears to be heterogeneous with diverse functional phenotypes that range from pro-inflammatory M1 phenotypes to immunosuppressive M2 phenotypes [reviewed in Tang and Le (2016)]. Given the highly complexity of microglial functions, the M1/M2 paradigm represents a simplified model to depict two polars of the inflammatory responses. This research topic in Frontiers in Aging Neuroscience has produced a highly informative collection of original research, reviews, mini-reviews, as well as technology report that cover comprehensive aspects related to microglial polarization. Researchers and clinicians have presented their work and views on the potential mechanisms of M1/M2 polarization that are critically associated with a broad spectrum of neurodegenerative diseases.

First, Subramaniam and Federoff provided a review on the molecules and signaling that modulate microglial polarization states in animal models and clinical trials of Parkinson's disease (PD), which supports targeting of microglial phenotypes as a novel therapeutic approach for PD. Porrini et al. used the nuclear factor-κB (NF-κB)/c-Rel deficient mice to investigate the dynamics of microglial polarization and mild inflammatory profiles that contribute to modeling late-onset Parkinsonism. Two original articles then focused on the potential effects of microglia phenotypes in Alzheimer's disease (AD). Hypoxia as a pivotal environmental factor has been demonstrated to aggravate AD via exacerbating Aβ and tau pathologies. Zhang et al. figured out that acute hypoxia promoted M1 polarization but suppressed M2 polarization in both AD transgenic mice and their wild type littermates, the process of which was associated with NF-κB induction through toll-like receptor 4 (TLR4). This study thus indicates the pathological role of hypoxia in the immunopathogenesis of AD. Mutations of DNAX-activating protein of 12 kDa (DAP12) and triggering receptors expressed on myeloid cell 2 (TREM2) have been risk genes for AD. In the study by Zhong et al. they demonstrated that TREM2/DAP12 complex suppressed the activity of JNK signaling to suppress lipopolysaccharides (LPS)-triggered M1 pro-inflammatory responses, thereby executing anti-inflammatory functions. Geloso et al. then analyzed the features and timing of M1/M2 microglial polarization in the transgenic mice of amyotrophic lateral sclerosis (ALS), and reviewed the preclinical approaches that may affect microglia phenotypes. Similarly, Yang et al. summarized current knowledge about M1/M2 microglial polarization and their corresponding signaling pathways in the pathogenesis and progression of Huntington's disease (HD).

Traumatic brain injury (TBI) can also trigger neurodegeneration and is a major risk factor for the development of dementia. Microglial polarization following TBI may contribute to the restoration of homeostasis in the brain. The study by Donat et al. reviewed the effects of microglia in TBI, including the analysis of their distribution, morphology and functional phenotypes over time in multiple experimental animal models and in humans. This may give an insight into the time course of microglial polarization at the injury site and the spreading of inflammation to other brain areas. It reminds us that ameliorating the inflamed milieu would be required to facilitate neuron rescue at the injury site and achieve long-term protection and recovery in TBI. And this is also applied for ischemic stroke. Liu et al. investigated the neuroprotective functions of dietary phytochemical curcumin in experimental stroke models, and demonstrated that curcumin post-treatment has a profound regulatory effect on microglial responses over time, promoting M2 polarization and suppressing pro-inflammation.

As an extension of the brain, retina is also affected in several neurodegenerative diseases. Ramirez et al. described the contribution in retina to microglia polarization in AD, PD, and glaucomatous neurodegeneration. Notably, those neuroinflammation in the retinal tissue might be of great use for the early diagnosis and monitoring of neurodegeneration. Obst et al. then reviewed the current understandings on the role of microglia in the pathogenesis of prion disease, which is a chronic neurodegenerative disorder characterized by the accumulation of scrapie prion protein PrP^Sc^. The cytokine profile is associated with both pro- and anti-inflammatory reactions, suggestive of a mixed inflammatory profile in the prion-diseased brain. The fact that different prion strains, disease stages, and detection techniques are also added into the complexity of inflammatory profiles in prion diseases.

Despite the simplified division of M1/M2 functionally polarization, modulation of microglial phenotypes has become a potential therapeutic approach for the treatment of neurodegenerative diseases. In light of this, Song and Suk reviewed recent studies of pharmacological modulators, especially the small-molecule compounds and their intracellular target proteins that participate in microglial polarization. Moreover, Pena-Altamira et al. also focused on nutritional approaches by the intake of food bioactive compounds such as carotenoids, phytosterols, and so forth, which may intervene microglial polarization, contributing to neuron survival and therefore improve cognitive aging impairment. Specifically, Roser et al. described how the inhibition of Rho kinase (ROCK) pathway can induce the microglia phenotype towards the beneficial “M2” and explored ROCK inhibition as a promising treatment option for PD and ALS. Atypical chemokine receptors (ACKRs), which belong to a small subset of chemokine receptors, were shown to limit inflammation through chemokine scavenge at the inflammatory sites. The review by Salvi et al. summarized the potential role of the ACKRs in neuroinflammation, particularly focusing on the microglial polarization and function. Moreover, Labandeira-Garcia et al. described the effects of the brain renin-angiotensin system (RAS) on microglial polarization. Angiotensin II (Ang II) acts through its type 1 receptor (AT1), leading to the NADPH-oxidase complex activation and giving rise to pro-oxidative and pro-inflammatory effects. In the opposite way, these effects are counteracted by Ang II/AT2 receptor signaling and Angiotensin 1-7/Mas receptor (MasR) signaling, which produce anti-inflammatory effects and promote M2 polarization.

It then comes to the technology section of studying microglia functions. The advancement of experimental cell-specific depletion methods in recent years has greatly boosted the microglial research. In the study by Lund et al. they reviewed and compared the different depletion approaches that have been used to eliminate microglia or monocyte-derived macrophages in a range of neurodegenerative disease such as ALS and AD, and discussed their prospects for immunotherapy. In the technology report by Noristani et al. the authors combined magnetic resonance imaging (MRI) and multiphoton to study microglia/monocytes alterations after spinal cord injury (SCI). Ex vivo diffusion MRI allows detailed examination of CNS tissues, while advances in clearing procedures allow detailed imaging of fluorescent-labeled microglia/monocytes at high resolution. Another critical method for studying microglia is definitely transcriptomes. Increasing studies have investigated microglial transcriptome remodeling over the course of pathogenesis and figured out the microglial response diversity to different diseases. Hirbec et al. therefore critically reviewed and compared the different methods developed to isolate microglia, and decipher microglial transcriptomes as well, including RNA-Seq and microarray, in multiple neuropathological conditions.

Aging is one of the most important risk factors for the onset and progression of neurodegenerative diseases, during which age-dependent and senescence-driven microglia function impairments have played essential roles. Spittau summarized the current knowledge of microglia phenotypes and functions in aged CNS, and also discussed the implications of age-related changes in microglia functions for the development of PD and AD. Specifically, Caldeira et al. investigated key-aging associated responses in microglia stimulated by Aβ in cell culture, with a reduced phagocytosis, migration and lower expression of inflammatory miRNAs over time. This study thus manifests the heterogeneous microglial responses along the progression of AD, and improves our understanding that therapeutic targeting microglia might differ from early to late stages. What is more, the crosstalk among glia, neural progenitor cells, and neurons also becomes dysfunctional during aging, along with harmful effects to dopaminergic neuron plasticity and repair. As reviewed by L'Episcopo et al. the aging-induced M1 pro-inflammatory responses disrupt the subventricular zone (SVZ) plasticity via PI3K-Wnt/β-catenin dysregulation, thereby impairing the neurogenic process.

The study by Walker et al. investigated the potential functions of colony stimulating factor-1 (CSF-1) and interleukin-34 (IL-34) in microglia derived from human autopsy AD brains. They showed that IL-34 induced primarily M1 responses similar to that of CSF-1, which is contrary to that in earlier work with rodent microglia. This discrepancy might come from either species difference or age difference, but it indeed reminds us that further studies shall also make the most use of human subjects to recapitulate the real AD pathology. Finally, Au and Ma described another type of microglia, bipolar/rod-shaped microglia, which is previously less studied mainly due to the lack of a well-established in vitro culture system. The spatial arrangement of bipolar/rod-shaped microglia is important for the reorganization and remodeling of neuronal and synaptic circuitry after CNS injuries. They then discussed the potential neuroprotective roles of bipolar/rod-shaped microglia, and the possibility of transforming ramified/amoeboid microglia into bipolar/rod-shaped microglia to promote CNS repair. This study will broaden our knowledge on microglia polarization at a very different angle.

However, as we mentioned above, the categorization of M1/M2 microglial polarization is quite simplified, since microglia may not display a significant bias toward either the M1 or M2 phenotype in a complicated microenvironment *in vivo*. With the advent of single-cell RNA-Seq, studies have shown that microglial transcriptome profiles are varied and context dependent, with regard to microglial polarization [reviewed in Ransohoff (2016), Colonna and Butovsky (2017)]. When defining microglial phenotypes, the combined variables such as age, neuropathological conditions and stages of disease will need to be accounted for, and genome-wide expression profiling technologies shall be further employed.

Overall, this collection is plenty of interesting articles that brought comprehensive understandings of microglial polarization in a range of neurodegenerative diseases, linking with aging-related alterations in microglia and technologies developed for microglial studies. We hope that this topic would give exciting insights into therapeutic approaches in the treatment of neurodegeneration, through targeting microglial polarization.

## Author contributions

The author confirms being the sole contributor of this work and approved it for publication.

### Conflict of interest statement

The author declares that the research was conducted in the absence of any commercial or financial relationships that could be construed as a potential conflict of interest.
